# Identification of the Mind Bomb1 Interaction Domain in Zebrafish DeltaD

**DOI:** 10.1371/journal.pone.0127864

**Published:** 2015-05-28

**Authors:** Gregory Palardy, Ajay B. Chitnis

**Affiliations:** Program in Genomics of Differentiation, Eunice Kennedy Shriver National Institute of Child Health and Human Development, National Institutes of Health, Bethesda, Maryland, United States of America; Texas A&M University, UNITED STATES

## Abstract

Ubiquitylation promotes endocytosis of the Notch ligands like Delta and Serrate and is essential for them to effectively activate Notch in a neighboring cell. The RING E3 ligase Mind bomb1 (Mib1) ubiquitylates DeltaD to facilitate Notch signaling in zebrafish. We have identified a domain in the intracellular part of the zebrafish Notch ligand DeltaD that is essential for effective interactions with Mib1. We show that elimination of the Mind bomb1 Interaction Domain (MID) or mutation of specific conserved motifs in this domain prevents effective Mib1-mediated ubiquitylation and internalization of DeltaD. Lateral inhibition mediated by Notch signaling regulates early neurogenesis in zebrafish. In this context, Notch activation suppresses neurogenesis, while loss of Notch-mediated lateral inhibition results in a neurogenic phenotype, where too many cells are allowed to become neurons. While Mib1-mediated endocytosis of DeltaD is essential for effective activation of Notch in a neighboring cell (in *trans*) it is not required for DeltaD to inhibit function of Notch receptors in the same cell (in *cis*). As a result, forms of DeltaD that have the MID can activate Notch in *trans* and suppress early neurogenesis when mRNA encoding it is ectopically expressed in zebrafish embryos. On the other hand, when the MID is eliminated/mutated in DeltaD, its ability to activate Notch in *trans* fails but ability to inhibit in *cis* is retained. As a result, ectopic expression of DeltaD lacking an effective MID results in a failure of Notch-mediated lateral inhibition and a neurogenic phenotype.

## Introduction

The Notch signaling pathway plays an evolutionarily conserved role in regulating interactions between adjacent cells in a wide range of developmental contexts [1] and its dysfunction can contribute to diverse problems including the development of cancer and the dysfunction of multiple organ systems [2, 3]. Unlike a number of signaling systems that allow cells to communicate over a distance via secreted ligands, both ligands and receptors in the Notch signaling pathway are at the cell surface and this signaling pathway primarily mediates interactions between adjacent cells.

The mature Notch receptor presents on the cell surface as a furin-cleaved extracellular domain (NECD) bound in a calcium-dependent manner to the remaining part, which includes an extracellular stub, a membrane-spanning region and an intracellular domain. Interaction of the Notch extracellular domain with the extracellular domain of a DSL (Delta, Serrate, Lag2) ligand, expressed on the surface of an adjacent cell, “activates” the Notch receptor. This interaction facilitates removal of the NECD fragment, which allows two sequential cleavage events that release the Notch Intracellular Domain (NICD) from the cell surface whereupon it translocates to the nucleus. There, NICD forms a complex with CSL (CBF1, Suppressor of Hairless, Lag-1) proteins and a number of other factors together with which it can drive transcription of target genes recognized by the CSL DNA-binding domain [4].

The mechanism by which DSL ligands interact to activate Notch is unusual; ubiquitylation-dependent endocytosis of the ligands is required for effective action of the Notch receptor. Though the precise mechanism by which endocytosis contributes to receptor activation remains only partially understood, a mechanical pulling and a recycling model have been proposed [5]. The mechanical pulling model suggests that ubiquitin-mediated endocytosis of a Notch ligand following its relatively strong binding to the Notch extracellular domain facilitates the separation of the NECD fragment from the remaining membrane tethered Notch receptor. Removal of the NECD exposes an ADAM metaloprotease cleavage site on the remaining Notch extracellular stub, and cleavage at this site facilitates a subsequent γ-secretase dependent intra-membranous cleavage of Notch, which results in the cytoplasmic release of the NICD fragment. Alternatively, or additionally, the recycling model suggests that endocytosis and subsequent recycling of Notch DSL ligands following ubiquitylation targets them to cell surface membrane compartments, including possibly lipid rafts, where they are able form more effective ligands for Notch.

The Neuralized and Mind bomb (Mib) families of RING E3 ligases mediate ubiquitylation and subsequent endocytosis of Delta and Serrate/ Jagged related DSL ligands in specific tissue contexts in various model systems [6–9]. In zebrafish, Mind bomb1-dependent DeltaD endocytosis depends on interaction of DeltaD with Notch receptors [10]. However, the mechanisms that regulate interactions between Mind bomb1 (Mib1) and DeltaD and subsequent endocytosis of DeltaD remain poorly understood. In an effort to clarify these mechanisms, we have tried to define the intracellular domain of DeltaD that is essential for interaction with Mib1 and its subsequent ubiquitylation by this E3 ligase in zebrafish.

## Methods

This study was carried out in strict accordance with the recommendations in the Guide for the Care and Use of Laboratory Animals of the National Institutes of Health. The Animal Study Proposal was approved by the Eunice Kennedy Shriver National Institute of Child Health and Human Health Animal Care and Use Committee (proposal #: 12–039). Collection of embryos following natural crosses of adults did not require surgery or anesthesia.

### Cell culture, Transfection, Immunoprecipitation and Western Blot Analysis

Interaction of Mib1 and DeltaD proteins was assayed by co-immunoprecipitation. COS 7 or HEK293T cells were grown at 37C/ 5%CO2 in DMEM supplemented with 10% fetal calf serum(FCS). Cells were transiently transfected with 2–3 ug plasmid DNA per 6 cm dish using Fugene6. The total amount of plasmid DNA was kept constant by adding an appropriate amount of the CS2+ vector plasmid. Cells were harvested at 18–24 hours post-transfection and lysed in modified RIPA buffer (50mM Tris-HCL, pH7.4, 150mM NaCl, 1% NP-40, 0.25% Sodium Deoxycholate) with a protease inhibitor cocktail (Roche complete). Clarified lysates were prepared by centrifugation and the supernatants pre-cleared with Protein A, G or A/G sepharose for 45 minutes. Precipitating antibodies (mouse anti-deltaD(zdd2), rabbit anti-Myc (Abcam or Cell Signalling) or mouse anti-HA (Covance) were added to the supernatants and incubated 2 hours at 4C. Protein A, G or A/G was added and the samples incubated an additional 45 minutes at 4C. The sepharose beads were washed 7x with modified RIPA buffer. The beads were boiled in NuPage LDS sample buffer and the eluted proteins loaded onto 4–12% NuPage bis-Tris acrylamide gels for electrophoresis then transferred to PVDF membrane. Membranes were washed in TBST (50mM Tris-HCL, pH 8.0, 150mM NaCl, 0.05% Tween-20) and blocked overnight in 5% nonfat milk in TBST. Blots were incubated with the following primary antibodies for 2 hours in 5% milk/ TBST; rabbit anti-Myc (Abcam or Cell Signalling), mouse anti-Myc (Stratagene 9E10), zdd2, mouse anti-FLAG (Sigma) or mouse anti-HA (Covance). Antibody dilutions were all 1:1000 with the exception of deltaD (1:2000). Blots were incubated with the secondary antibodies; goat anti-mouse,—rabbit or—rat horseradish peroxidase (Jackson Immunoresearch) at 1:10,000 dilution for 1 hour in 5% milk/TBST. Visualization was by chemiluminescent detection (Pierce West-Pico or GE Healthcare ECL-Plus).

### Immunocytochemistry and Antibody Internalization Assay

In vitro expression of the DeltaD constructs with and without Mib1 was assayed by immunocytochemistry. COS7 cells were grown on coverslips in 6-well dishes and transfected with Fugene6. At 24 hours post-transfection, cells were washed with cold PBS supplemented with calcium and magnesium at 4C then incubated on ice for 30’ with cold DMEM containing zdd2 (1:2000). Cells were washed extensively with cold PBS(Ca, Mg) and returned to 37C with warm DMEM/ 10% FCS for an additional 30’. Cells were washed in PBS then fixed in 4% paraformaldehyde, 15’ at 4C then 10’ at RT. Coverslips were washed with PBS (Ca, Mg) and blocked overnight in 10% goat serum in PBS. Coverslips were washed and placed on drops of PBS/2% goat serum/0.2% saponin with rabbit anti-Myc (1:1000) for 1 hour at RT. Coverslips were washed and the procedure repeated with the secondary antibodies, goat anti-mouse Alexa 488(1:250 dilution) or goat anti-rabbit Alexa 546(1:250 dilution) and DAPI. Coverslips were washed and mounted with Fluoromount G on slides then imaged on a Zeiss LSM510 confocal microscope.

### Plasmid constructs and mRNA preparation

Myc-Mib1, DeltaD-HA, DeltaD∆A-D-HA and HA-Ubiquitin in pCS2+ have been previously described [9]. The full length DeltaD and intracellular truncations ∆D, ∆C-D, and ∆B-D and ∆A-D in pCDNA3.1 were a gift from Julian Lewis. The ∆A, ∆B, ∆C, ∆D, NN, KK, and NN/KK constructs in pCS2+ were generated by inverse PCR mutagenesis. For mRNA preparation for injection, the DeltaD constructs in pCS2+ were linearized with PspOM I and transcribed with SP6 RNA polymerase using mMessage Machine kit (Ambion).

### mRNA Injection and Whole Mount In Situ Hybridization

Adult zebrafish were housed in an AAALAC accredited facility maintained at a 14 hour/10 hour day/night cycle. DeltaD proteins were overexpressed in zebrafish embryos by mRNA injection. Various DeltaD and ß-Galactosidase mRNAs were co-injected into 2-cell stage zebrafish AB*embryos. At 3–4 somite stage, embryos were dechorionated and fixed for 1 hour at room temperature in 4% PFA. Embryos were washed in PBT and incubated in X-Gal buffer (35 mM K_4_Fe(CN)_6_*3H_2_O, 35 mM K_3_Fe(CN)_6_, 2 mM MgCl_2_, 0.1% Tween-20, 0.01% Na Deoxycholate) overnight at 4C with 1 mg/ml X-Gal. After development of b-Galactosidase staining embryos were washed and fixed overnight in 4% PFA. Whole mount in situ hybridization followed standard protocol with previously established anti-sense *huC* probes [11].

## Results

### Identification of the Mib1 interaction domain in DeltaD

**Fig 1:**
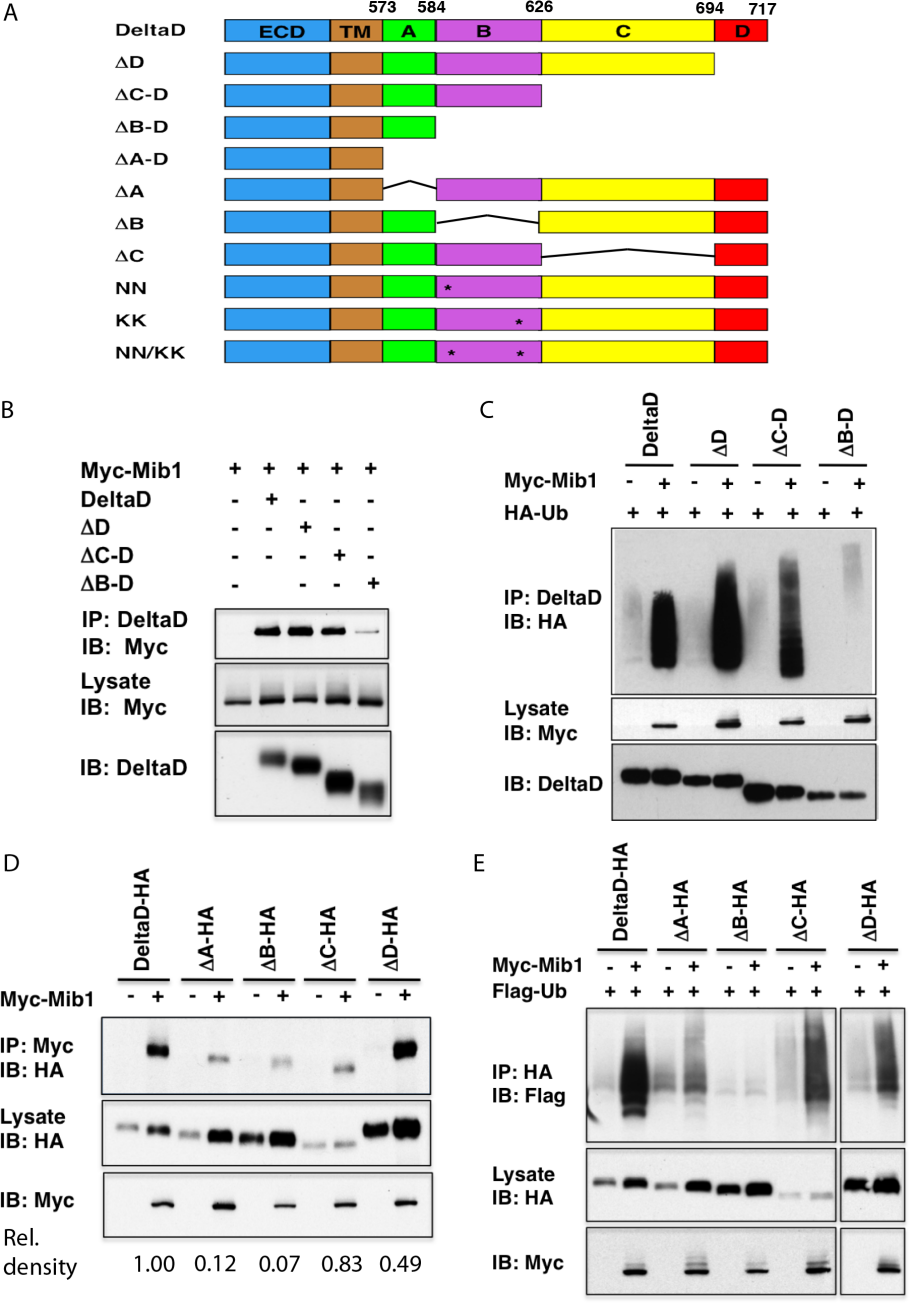
Identification of the *Mib1*-interacting domain (MID) in the Notch ligand DeltaD. (A) DeltaD deletion constructs and point mutations. Asterisks represent relative positions of NN and KNxNKK motifs. (B) Mib1 does not interact effectively with DeltaD ∆B-D. Myc-Mib1 was co-immunoprecipitated with full-length DeltaD and truncation mutants (∆D, ∆C-D, ∆B-D) using zdd2 antibody (Ab) and detected with anti-Myc Ab. (C) ∆B-D is not effectively ubiquitylated by Mib1. Full-length and DeltaD truncation mutants, co-transfected with HA-ubiquitin (HA-Ub), with and without Myc-Mib1, were immunoprecipitated with zdd2 Ab and immunoblotted with anti-HA Ab to detect ubiquitylated DeltaD. (D) Delta ∆A (∆A) and Delta ∆B (∆B) interact poorly with Mib1. HA-tagged DeltaD and deletion constructs co-transfected with and without Myc-Mib1 are immunoprecipitated with anti-Myc Ab and detected with anti-HA Ab. Relative density of IP anti-Myc band normalized to lysate anti-HA band. (E) ∆B is not effectively ubiquitylated by Mib1. DeltaD-HA and deletion constructs are immunoprecipitated with anti-HA Ab to detect total ubiquitylated DeltaD with and without Myc-Mib1.

cDNAs encoding four successive C-terminal truncations of DeltaD (Fig 1A) were initially utilized to identify the domain required for interaction of DeltaD with Mib1: DeltaD ∆D (with terminal amino acids (aa) 694 to 717 deleted), DeltaD ∆C-D (with aa 626 to 717 deleted), DeltaD ∆B-D (with aa 584 to 717 deleted) and DeltaD ∆A-D (with aa 573 to 717 deleted). HEK293T cells were transiently transfected with the full-length or truncated forms of DeltaD together with Myc-Mib1. DeltaD was then immunoprecipitated with a DeltaD specific monoclonal antibody, zdd2, which recognizes the extracellular domain of DeltaD [12], and co-immunoprecipitation of Mib1 was assessed with an anti-Myc antibody. While Myc-Mib1 was co-immunoprecipitated with DeltaD ∆D and DeltaD ∆C-D, it was not co-immunoprecipitated with DeltaD ∆B-D suggesting that the B domain of DeltaD is necessary for the interaction with Mib1 (Fig 1B). It should be noted that, for reasons that seem unclear, DeltaD lacking the domains A-D (DeltaD ∆A-D) was found to non-specifically co-immunoprecipitate with the anti-Myc antibody in this assay and could not be used to make any meaningful conclusion about interactions with Mib1 (data not shown). To determine if the B domain is required for ubiquitylation of DeltaD by Mib1, the truncated forms of DeltaD were co-transfected with Mib1 and an HA-tagged Ubiquitin. DeltaD constructs, immunoprecipitated with zdd2 were immunoblotted with an anti-HA antibody to determine if they had been effectively ubiquitylated by Mib1. Again, while full length DeltaD, DeltaD ∆D and DeltaD ∆C-D were effectively ubiquitylated, DeltaD ∆B-D was not, suggesting that region B in the intracellular domain of DeltaD is required for interaction with Mib1 and for ubiquitylation by it (Fig 1C).

To further specify the interaction domain, HA-tagged versions of full length DeltaD (DeltaD-HA) or with the A (aa 573–584), B (aa 585–625), C (aa 626–694) or D (694–717) intracellular domains specifically deleted (∆A-HA, ∆B-HA, ∆C-HA, ∆D-HA) were then co-transfected with or without Myc-Mib1 to confirm the interaction domain. Immunoprecipitation of Myc-Mib1 with an anti-Myc antibody followed by immunoblotting with an anti-HA antibody showed relatively low levels of Myc-Mib1 associated with ∆A-HA, ∆B-HA and ∆C-HA, while similar levels were pulled down with ∆D-HA and full length DeltaD-HA. However, the relatively low levels of Myc-Mib1 associated with ∆C-HA were associated with similarly low levels of ∆C-HA in the lysate. This suggested that it was the loss of region A or B in the DeltaD intracellular domain that resulted in the most reduction in interaction with Mib1 (Fig 1D), while loss of region C in the intracellular domain appeared to produce a relatively unstable form of DeltaD. After normalizing for the amount of DeltaD protein in the lysate, a comparison of the amount Myc-Mib1 pulled down, suggested that loss of the B region in the DeltaD intracellular domain caused the most reduction in interaction between DeltaD and Mib1. (Fig 1D). Consistent with these observations, the specific deletion of region A or B also resulted in a decrease in Mib1-mediated ubiquitylation, with loss of the region B causing the greater decrease (Fig 1E). On the other hand, specific loss of region C was associated with relatively high levels of ubiquitylation, and, as indicated by the relatively low levels in the lysate, a less stable protein.

From the co-immunoprecipitation and ubiquitylation assays for the DeltaD truncations and deletions, Region B was found to constitute a major interaction domain of Mib1 and was chosen for further analysis. It should be noted that deletion of Region A also reduced the ability to interact with Mib1, however, its significance was not pursued in this study.

### A conserved motif within the Mib1 interaction domain of DeltaD is critical for interaction and ubiquitylation of DeltaD

**Fig 2:**
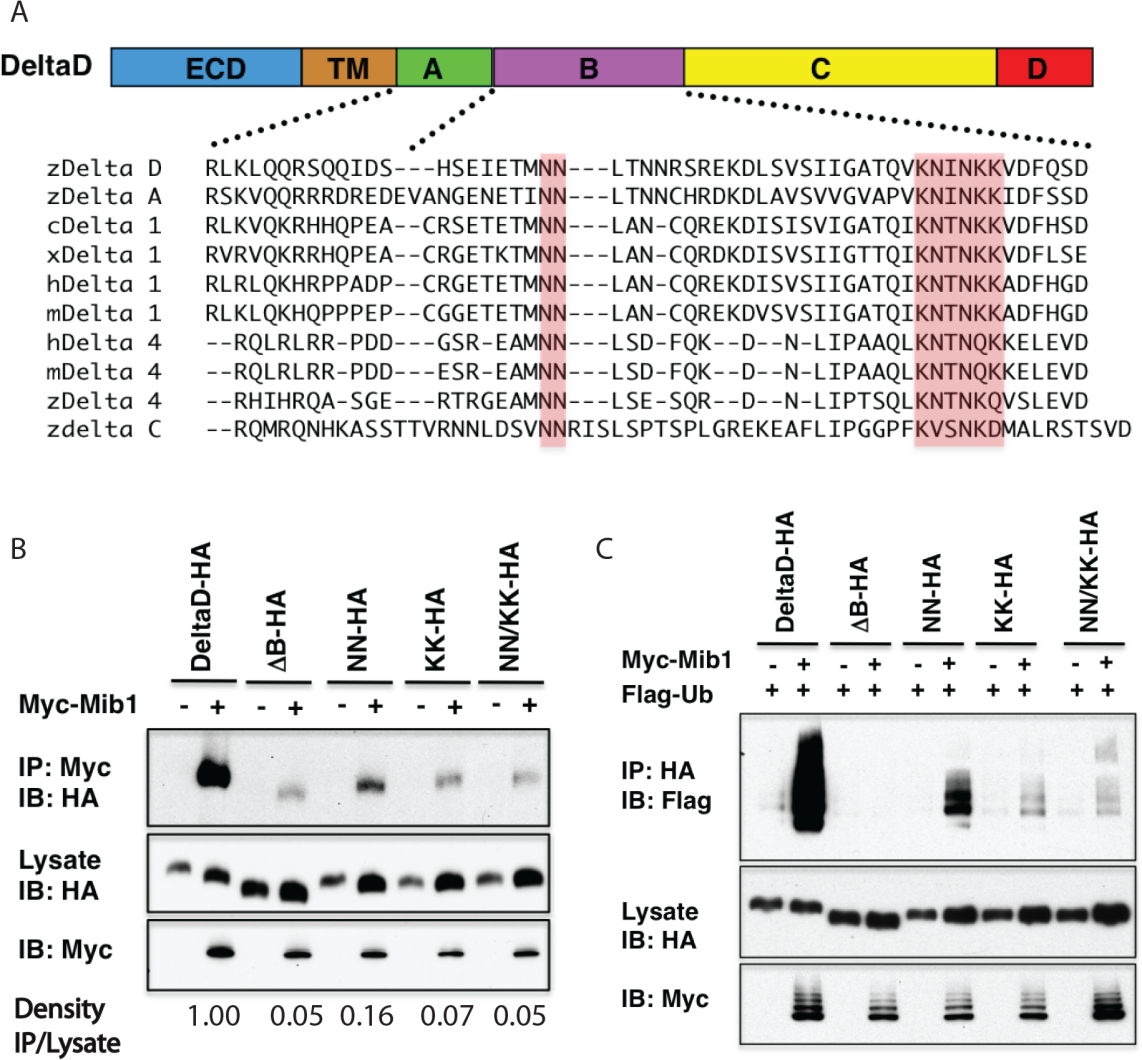
Identification of critical residues in the Mib1-Interacting Domain (MID). (A) Conserved amino acids (pink shading) in the putative *Mib1*-Interacting Domain in Delta ligands. (B) KK and NN/KK mutants do not significantly interact with mind bomb. DeltaD-HA, ∆B and point mutation (NN, KK and NN/KK) constructs are co-immunoprecipitated with Myc-Mib1 using anti-Myc Ab and detected with anti-HA Ab. (C) KK and NN/KK mutants are not significantly ubiquitylated by Mib1. DeltaD-HA, ∆B and point mutation constructs co-transfected with Flag-Ubiquitin (Flag-Ub) with and without Myc-Mib1 are immunoprecipitated with anti-HA Ab and detected with Anti-Flag Ab to assay ubiquitylation of full length and mutant forms of DeltaD.

ClustalW2 alignment (Larkin et. al., 2007) of region B in DeltaA with homologous sequences in xenopus, chicken, mouse and human Delta Notch ligands revealed two conserved motifs, a di-asparagine pair (NN) and a lysine-rich region (KNxNKK) (Fig 2A). Three sets of mutations in region B mutants, now referred to as a Mib1-interacting domain (MID), were generated to assess the contribution of the conserved regions to interaction with Mib1 and its ability to ubiquitylate DeltaD; DeltaD-NN (N591A / N592A), DeltaD-KK (K614A / N615A / N617A / K618A / K619A) and a combination of these, DeltaD-NN/KK. Transient transfection and co-immunoprecipitation revealed that mutant forms of DeltaD (DeltaD-KK, DeltaD-NN/KK) that included substitutions in the lysine-rich domain resulted in the loss of interaction and ubiquitylation by Mib1 as seen with the ∆B deletion (Fig 2A, 2B and 2C). The Delta-NN mutant also showed reduced interaction and ubiquitylation with Mib1, however, the reduction was less than with the mutant KNxNKK domain. Together, these observations suggested that critical residues for interaction and ubiquitylation are likely contained within the KNxNKK residues, though the NN residues may play an accessory role. It should be noted that even the NN/KK mutant did not fully abrogate all Mib1-mediated ubiquitylation as seen in the ∆B deletion mutant indicating there may be some other residues contributing to the interaction.

### The NN and KNxNKK motifs are necessary for efficient internalization of DeltaD by Mib1

**Fig 3:**
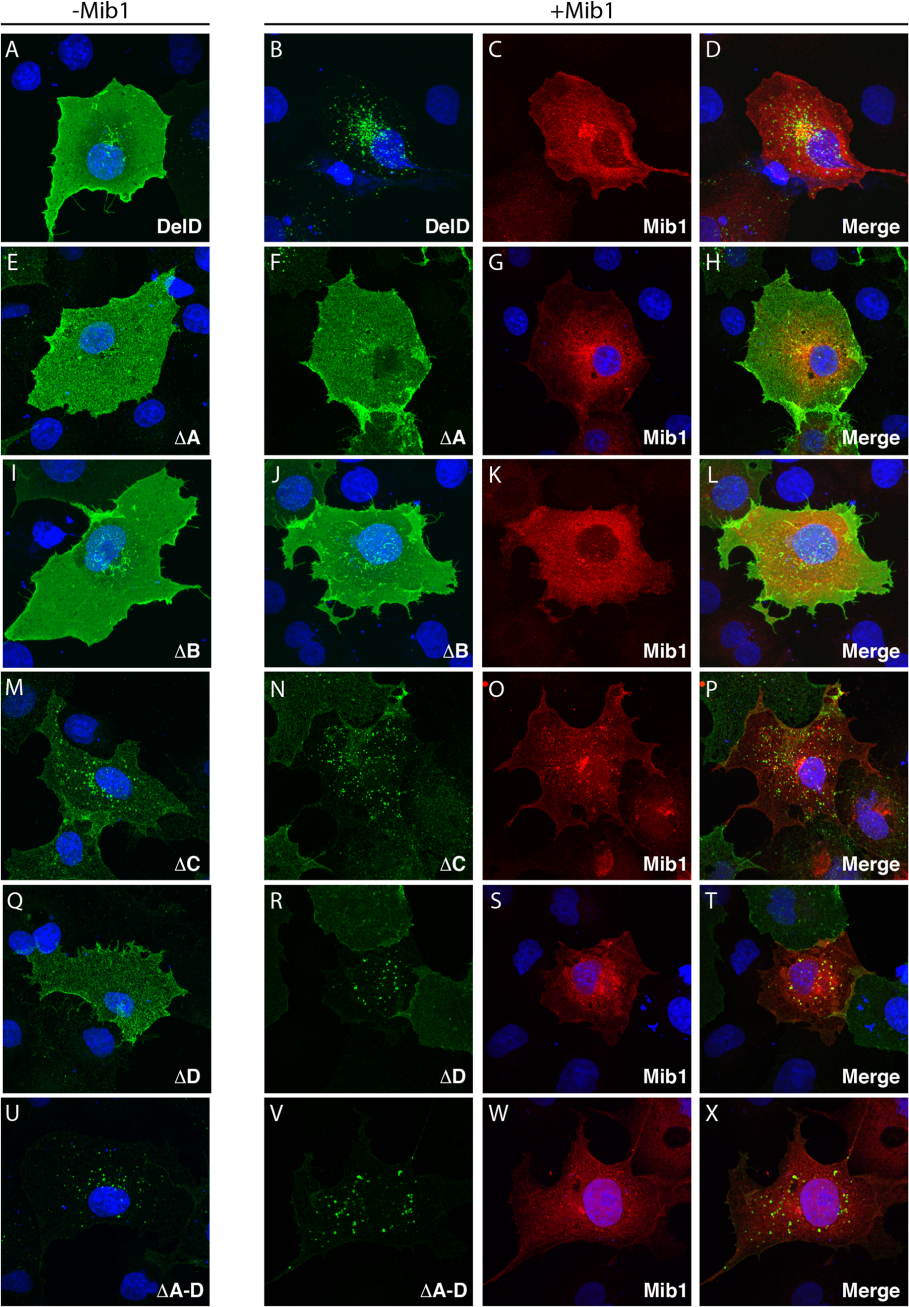
Endocytosis of DeltaD deletion mutants. (A,E,I,M,Q,U) Distribution of zdd2 (green) in COS7 cells transfected with DeltaD (A) or DeltaD ∆A, ∆B, ∆C, ∆D or ∆A-D deletion mutants (E, I, M, Q, U). Surface DeltaD was first labelled by incubation with zdd2 at 4°C for 30’ then, following washout of unbound zdd2, internalization was allowed for 30’ at 37°C. Nuclei were labelled with DAPI (blue). (B-D, F-H, J-L, N-P, R-T, V-X) Distribution of zdd2 (green) in COS7 cells co-transfected with DeltaD constructs and Mib1 (red) following internalization as described above. Each set of 3 panels, respectively, shows distribution of the DeltaD construct (green), Myc-Mib1 (red)/nuclei (blue), and the merged image. See materials and methods for details.

**Fig 4:**
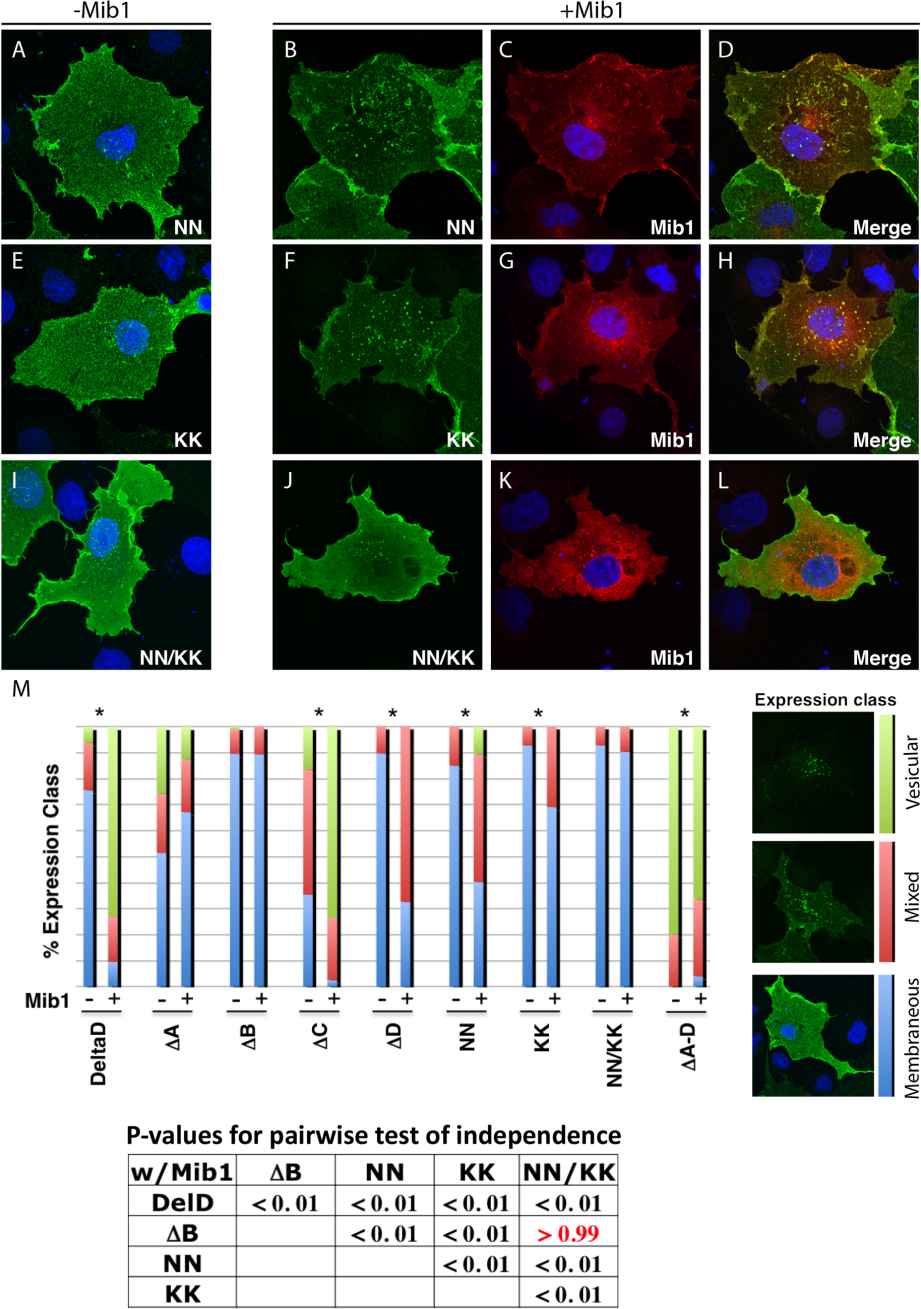
Endocytosis of DeltaD point mutants. Distribution of zdd2 (green) in COS7 cells transfected with DeltaD-NN (A), DeltaD-KK (E) and DeltaD-NN/KK following the internalization protocol described in Fig 3. Nuclei were labelled with DAPI (blue). Distribution of zdd2 (green) in COS7 cells co-transfected with DeltaD-NN (B-D), DeltaD-KK (F-H), DeltaD-NN/KK (J-L) and Mib1 following internalization as described above. Each set of 3 panels, respectively, shows distribution of the DeltaD construct (green), Myc-Mib1 (red)/nuclei (blue), and the merged image. (M) Summary of expression classes found in DeltaD full length, deletion and point mutants assayed following the internalization protocol described above. P-values for pairwise comparison based on Fisher’s Exact test of independence. P >. 05 does not meet the criteria for the Null hypothesis that pairs contain an equivalent distribution of expression classes.

An internalization assay with the zdd2 monoclonal antibody, raised against the extracellular domain of DeltaD was performed to compare the ability of Mib1 to determine ubiquitylation-dependent endocytosis of wild-type or mutant DeltaD in transiently transfected COS7 cells (Fig 3 and quantification in Fig 4). In this assay, 24 hours after transfection, cells were exposed to the zdd2 antibody for 30 minutes at 4°C so the antibody could bind to DeltaD on the cell surface. Then the cells were washed and shifted to 37°C for 30 minutes to allow endocytosis of the bound complex. The cells were then fixed and localization of the bound mouse zdd2 monoclonal antibody was assayed with a fluorescent anti-mouse secondary antibody.

In the absence of Mib1 co-transfection, most of the zdd2 associated with DeltaD remained on the cell surface (Fig 3A). On the other hand, in cells with DeltaD co-transfected with Mib1, zdd2 was typically in internalized vesicles, associated with endocytosed Mib1 (Fig 3B). However, in some cases zdd2 was seen in a pattern consistent with it being distributed both on the cell surface and in intracellular vesicles. To quantify how Mib1 influences the distribution of wild-type DeltaD or its specific mutated forms, cells were classified and quantified based on whether DeltaD, as revealed by the distribution of zdd2, was primarily on the cell surface (Class 1, blue bar), both on the surface and in intracellular vesicles (Class 2, red bar) or primarily in intracellular vesicles (Class 3, green bar), when DeltaD was transfected with or without Mib1 (Fig 4). As noted above, in cells not transfected with Mib1, most cells have DeltaD on the surface (Class 1). However, in cells co-transfected with Mib1 there was a significant change in the distribution of DeltaD, as it shifted to intracellular vesicles (Class 3) in most cells. In contrast, DeltaD-∆A and DeltaD-∆B showed little change in zdd2 distribution when cells were co-transfected with Mib1 and most DeltaD remained on the cell surface (Class 1), consistent with these domains being required for interaction with Mib1 and/or for Mib1-mediated endocytosis. On the other hand, though cells transfected with the ∆C mutant initially showed a greater proportion of both intracellular vesicles and surface expression of zdd2 (Class 2), addition of Mib1 clearly increased the number of cells with zdd2 in intracellular puncta (Class 3) (Fig 3D). The ∆D mutant had a predominantly cell surface distribution (Class 1) and, as with full length DeltaD, there was a significant increase in internalized DeltaD in the presence of Mib1. However, unlike with full length DeltaD, where co-transfection with Mib1 primarily results in an increase in the number of cells with zdd2 in intracellular vesicles (Class 3), co-transfection with Mib1 resulted primarily in an increase of cells with zdd2 on both the cell surface and in intracellular vesicles (Class 2). In the ∆A-D mutant, where all four intracellular domains were removed, most DeltaD was primarily localized to intracellular vesicles (Class 3) with little to no expression on the surface. There was no obvious increase in the intracellular distribution of ∆A-D in the presence of Mib1, as most of it was already in intracellular vesicles in the absence of Mib1.

Though both the DeltaD-NN and DeltaD-KK mutants had reduced interactions with Mib1 in pull-down and ubiquitylation assays, co-transfection of these mutants with Mib1, nevertheless significantly increased the fraction of cells with internalized DeltaD, suggesting that interactions with Mib1 were not compromised enough to prevent Mib1-mediated internalization (Fig 4). On the other hand, co-transfection of the DeltaD-NN/KK double mutant with Mib1 did not significantly increase the fraction of cells with internalized DeltaD compared to the wild type. This suggests that the di-asparagine pair and the lysine-rich region of the deltaD intracellular domain are, together, critical for Mib1-dependent internalization. Interestingly, the complete deletion of the DeltaD intracellular domain (∆A-D) showed significant internalization without Mib1 present (Fig 3B).

### Over-expression of NN/KK mutants in zebrafish fails to suppress neurogenesis while recapitulating the neurogenic phenotype of DeltaD ∆A-D

**Fig 5:**
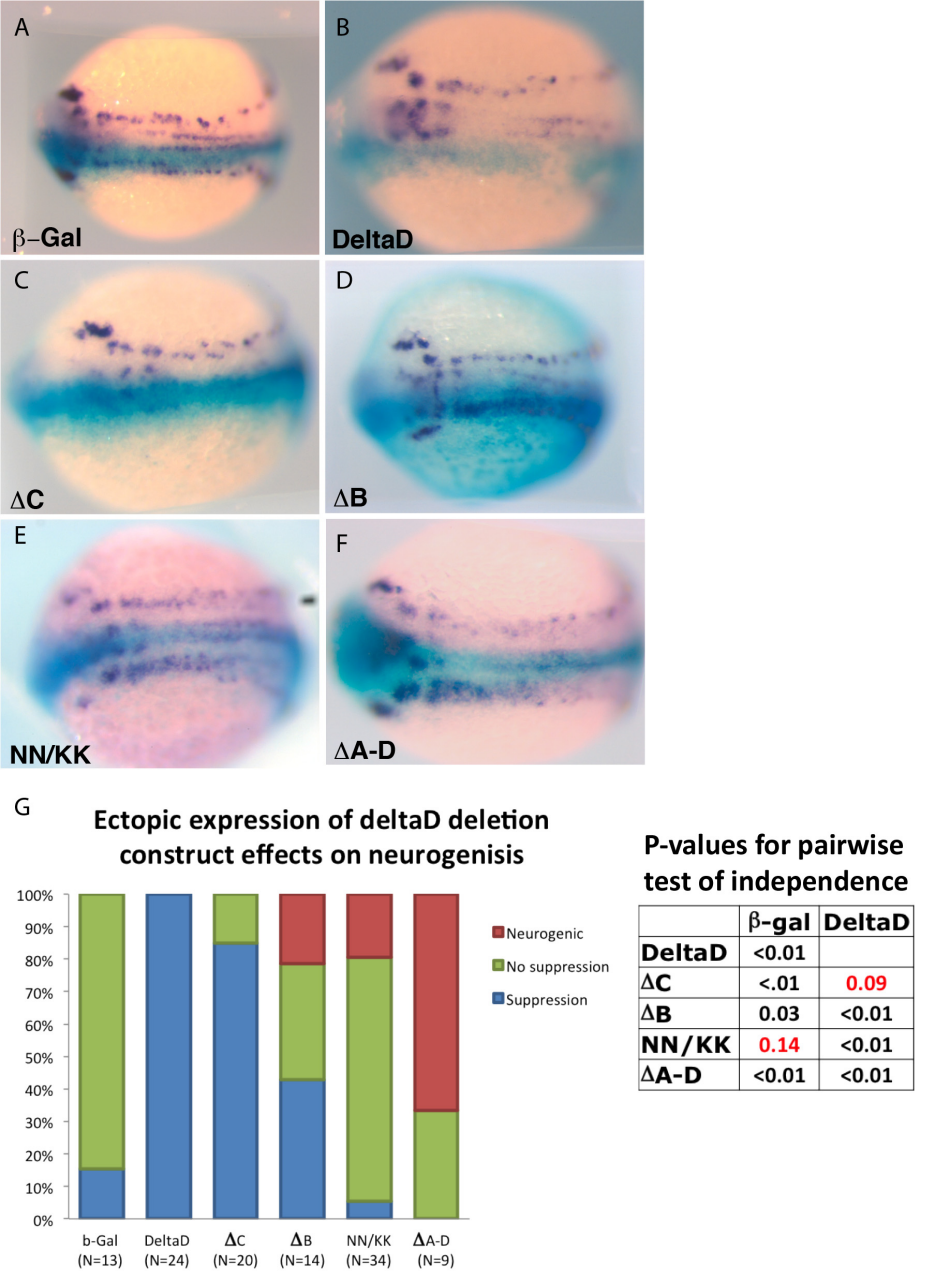
Ectopic expression of deltaD deletion and point mutant recapitulates neurogenic phenotype of DeltaD (∆A-D). (A) The prospective distribution of neurons revealed by the distribution of *huC* as revealed by *in situ* hybridization probe (purple) in control embryos injected with only *ß-galactosidase* mRNA. (B-F) *huC* in embryos co-injected with *ß-galactosidase* and *deltaD* (B), *deltaD-∆C* (C), *deltaD ∆B* (D), *deltaD NN/KK* (E) or *DeltaD ∆A-D* (F) mRNA. Distribution of ectopic mRNA injected in one cell at the two-cell stage revealed by X-Gal distribution (blue). Dorsal view, rostral to the left. Embryos are at approximately the 3 somite stage. (G) Quantification of the effect of ectopic expression of mRNA encoding various forms of DeltaD on the distribution of early neurons. Red indicates fraction with a neurogenic phenotype (increased density of neurons), Green—fraction with no obvious effect on neuron density, Blue- fraction with suppression of neurogenesis (reduced neuron density). P-values for pairwise comparison based on Fisher’s Exact test of independence. P >. 05 does not meet the criteria for the Null hypothesis that pairs contain an equivalent distribution of phenotype classes.

To test the in vivo function of residues implicated in Mib1 interaction, ubiquitylation and ligand internalization, mRNA encoding DeltaD, DeltaD deletion or point mutants was asymmetrically injected into two cell stage zebrafish embryos. As previously described, overexpression of DeltaD mRNA results in a reduction of neurons in the neural plate at 3 somite stage as evidenced by a reduction in the cells expressing HuC (Fig 5A and 5B and quantification in G), a marker for differentiating neurons [9]. This is due to inhibition of neural differentiation following lateral inhibition mediated by Notch signaling. Similarly, the DeltaD-∆C deletion was also capable of suppressing neurogenesis (Fig 5C and 5G). However, consistent with the relative instability of DeltaD-∆C, its effects were weaker than DeltaD; while ectopic expression of *deltaD* mRNA caused strong suppression of neurogenesis, injection of similar levels of *deltaD-∆C* mRNA caused weak suppression of neurogenesis (S1 Chart). In contrast to DeltaD and DeltaD-∆C, overexpression of DeltaD-∆B (Fig 5D and 5G) resulted in either no neural suppression or a weak neurogenic phenotype, as was previously shown with over-expression of a form of *Xenopus* Delta1 that lacks most of its intracellular domain [13] or with over expression of DeltaD ∆A-D (Fig 5F and 5G), in which, similarly, most of the intracellular domain is deleted. Injection of the NN/KK mutant recapitulated effects of the ∆B mutant injection and again resulted in a neurogenic phenotype in some embryos (Fig 5E and 5G).

## Discussion

In this study we have defined an intracellular domain of the Notch DSL ligand, DeltaD, which we now call the MID (Mib1-interacting Domain), that is essential for interaction with the RING E3 ligase Mib1. This domain, which extends from amino acids 585–625, was found to be essential both for ubiquitylation of DeltaD by Mib1 and for its ability to mediate effective Notch signaling, as assayed by its ability to suppress differentiation of neurons when ectopically expressed in the embryo.

A comparison of this stretch of 160 amino acids to other zebrafish Delta homologues and to Chick, *Xenopus*, human and mouse Delta1 and Delta4, revealed at least two stretches of well-conserved amino acid sequences, a di-asparagine pair (NN) at position 591–592 and a lysine rich region (KNxNKK) at position 614–619. Substitutions within these conserved amino acids revealed that while both contribute to effective DeltaD function, alteration of the KNxNKK amino acids caused a much greater reduction in interaction and ubiquitylation by Mib1. On the other hand, mutations in both conserved domains were required to significantly reduce Mib1-mediated internalization of DeltaD

When Delta interacts with Notch in a neighboring cell (in trans), endocytosis of Delta, bound to the Notch extracellular domain, facilitates activation of Notch in the neighbor. On the other hand, when the Delta interacts with Notch in the same cell (in cis), it does not result in activation; instead it results in *cis* inhibition of Notch signaling. In this context, inhibition of Notch is not dependent on Mib1-mediated endocytosis of Delta, it only requires interaction between the extracellular domains of Delta and Notch, as this reduces the possibility of interaction of Notch in *trans* with Delta in a neighboring cell, which could have resulted in Notch activation.

Lateral inhibition mediated by Notch signaling inhibits expression of factors required for differentiation of early neurons [9, 13, 14]. In this context, ectopic expression of DeltaD results in exaggerated activation of Notch and inhibition of early neurogenesis. On the other hand, as previously shown previously with XDelta1^STU^, a form of *Xenopus* Delta1 lacking its intracellular domain [13, 14], ectopic expression of DeltaD∆A-D, results in inhibition of Notch signaling, failure of lateral inhibition, and a neurogenic phenotype, where too many cells are allowed to prematurely differentiate as neurons. This is because in absence of its intracellular domain and Mib1-mediated endocytosis, DeltaD cannot activate Notch in *trans* but it is still capable of *cis* inhibition of Notch in the cell in which both Delta and Notch are expressed, as *cis* inhibition is dependent on interactions between the extracellular domains of Notch and its ligands [13–18]. Consistent with this expectation, in this study we have shown that the forms of DeltaD that lack intracellular domains required for effective interaction with Mib1 (Delta∆A-D, DeltaD∆B and DeltaD(NN/KK)), inhibit Notch signaling and induce a neurogenic phenotype when expressed at high enough concentrations.

While DeltaD is predominantly distributed in intracellular vesicles in the embryo, it is prominently expressed on the cell surface when transfected in COS7 cells because its trafficking to the cell surface exceeds endogenous mechanisms that would promote its subsequent endocytosis. In this context, co-transfection of Mib1 results in a clear shift in the cellular distribution of DeltaD by reducing the fraction of cells with predominantly cell surface distribution of Delta and increasing the fraction with internalized DeltaD in intracellular vesicles. The elimination of intracellular domains or motifs in DeltaD, required for interaction with Mib1, as in DeltaD∆B or DeltaD-NN/KK, results in forms of Delta whose cellular distribution cannot be as effectively altered by co-expression of Mib1. Though its function was not extensively examined in this study, elimination of the most proximal intracellular domain in DeltaD-∆A, also resulted in a form of DeltaD, whose co-transfection with Mib1 did not increase the fraction of cells with internalized DeltaD. Juxta-membrane domains also have been shown to regulate trafficking in other receptors [19–24] and its potential role in DeltaD will be investigated in future studies.

DeltaD-∆D, which has lost a terminal PDZ binding domain, was effectively internalized following co-transfection of Mib1. Nevertheless, compared to DeltaD, a smaller fraction of cells co-transfected with Mib1 had DeltaD-∆D predominantly in intracellular vesicles and a greater fraction had it in both intracellular vesicles and on the cell surface. This suggests that while the PDZ domain does not directly determine interactions with Mib1 it may nevertheless influence the efficiency of internalization and/or increase the recycling of DeltaD back to the surface.

Finally, loss of the C domain significantly reduced the amount of DeltaD-∆C on the cell surface and increased Mib1-independent internalization, nevertheless co-transfection with Mib1 increased the fraction of internalized DeltaD. Since DeltaD-∆A-D is unable to interact with Mib1 it was expected that it would primarily be localized on the cell surface, however, as with DeltaD-∆C, most DeltaD-∆A-D was associated with internalized vesicles. This is consistent with absence of the C domain in both DeltaD-∆C and DeltaD-∆A-D mutants determining exaggerated internalization by some Mib1-independent mechanism. However, in contrast to what is seen with DeltaD-∆C, co-transfection of DeltaD-∆A-D with Mib1 did not increase the fraction with internalized DeltaD-∆A-D as this form of DeltaD is incapable of interacting with Mib1. The mechanism that increases Mib1-independent internalization of DeltaD in the absence of the C domain is not known and remains a subject for future investigations.

We have identified a putative Mib1-interacting domain (MID) in the intracellular domain of DeltaD and have shown that it is required for both interaction and ubqiuitylation by Mib1. Similar analysis has been done to identify the Mib1-interacting domain in *Drosophila* Delta. Though there is significant homology in the extracellular domain of vertebrate and insect DSL ligands, overall sequence homology in the intracellular domain is poor, making it difficult to identify critical conserved domains. Domains in *Drosophila* Delta critical for interactions with Mib1 were characterized after four conserved regions (ICD1, ICD2, ICD3 and ICD4) in intracellular domain of Delta in a number of insects were defined and of these ICD2 is essential for interaction with and ubiquitylation by Mib1 [25]. Similarly, we used a comparison of the intracellular domain of the vertebrate Delta homologues to define potentially critical domains in DeltaD. Interestingly, these independent studies have now helped identify homologous domains in both vertebrate and insect Delta homologues. While the Daskalaki study identified a conserved domain NIIKNTWD at position 680–687 in *Drosophila* Delta as being critical for interaction and ubiquitylation by Mib1, our study identified a conserved domain KNINKK at position 614–619 to be one of two domains essential for effective interaction of DeltaD with Mib1. Sequence comparison centered around these independently identified Mib1 interaction domains now reveals a broader domain of conservation at positions 680–691 in *Drosophila* Delta (N**IIKNTWDKSVN**) and positions 606–617 in zebrafish DeltaD (S**IIGATQVKNIN**). Further comparison of corresponding conserved sequence in insect (*Apis*, *Pediculus*, *Bombyx*, *Tribolium*, *Periplaneta*, *Aedes*) Delta and vertebrate (zebrafish, chick, *Xenopus*, human and mouse) Delta1 homologues helped define the conserved motif ϕ [X]_5_ K [X]_2_ N (shown in **bold** above). The broader functional significance of this conserved sequence will need to be explored in future studies.

Our study also identified a conserved di-asparagine pair (**NN**) at position 591–592 in DeltaD within the MID. This di-asparagine motif appears related to a previously identified larger conserved domain in DSL ligands (Glittenburg et al). The study by Glittenburg et al showed that Serrate/Jagged homologues have a conserved motif [E/D][E/D]X_[_ _2__–_ _3__]_ **NN**X_5_NX_[_ _3__–_ _5__]_NP[L/I] and a mutant form of Serrate that lacks this motif lose the ability to trans-activate Notch. Delta homologues have a related motif [E/D]X_[_ _2__–_ _4__]_ **NN**[L/I]. Our study now shows that the conserved di-asparagines within this broader motif are required for effective interaction, ubiquitylation and internalization of DeltaD by Mib1. Together these observations suggest that despite poor overall similarity in the intracellular domain of DSL ligands short conserved domains that are functionally important have now been identified. We expect the identification of these critical Mind bomb interaction domains will provide an important starting point for future studies examining the mechanisms that regulate interaction of Mib1 and Delta in both vertebrates and invertebrates.

## Supporting Information

S1 Chart Effects of expression of DeltaD mutants on neurogenesis. Quantification of effects of injecting mRNA encoding DeltaD, DeltaD ∆C, DeltaD ∆B, DeltaD ∆A-D or DeltaD NN/KK mutants on neurogenesis. Embryos were scored as having a neurogenic (increased number /density of huC expressing cells) phenotype, or having no suppression, weak suppression or strong suppression of neurogenesis.
(TIFF)
