# Supplementary figures and images for: Identification of the Mind Bomb1 Interaction Domain in Zebrafish DeltaD
Source: PLoS One. 2015 May 28;10(5):e0127864. doi: 10.1371/journal.pone.0127864 (PMC4447371; doi:10.1371/journal.pone.0127864)

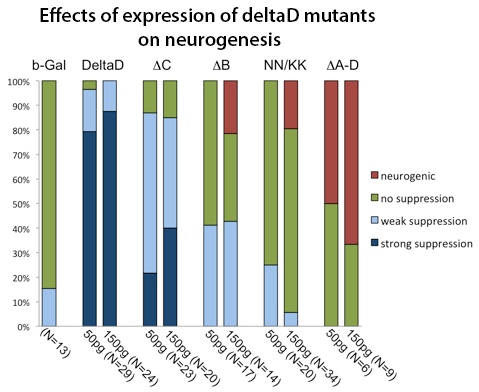

Supplement: S1 Chart — Quantification of effects of injecting mRNA encoding DeltaD, DeltaD ∆C, DeltaD ∆B, DeltaD ∆A-D or DeltaD NN/KK mutants on neurogenesis. Embryos were scored as having a neurogenic (increased number /density of huC expressing cells) phenotype, or having no suppression, weak suppression or strong suppression of neurogenesis. (TIFF) [file pone.0127864.s001.tiff]
